# Efforts Made to Eliminate Drug-Resistant Malaria and Its Challenges

**DOI:** 10.1155/2021/5539544

**Published:** 2021-08-30

**Authors:** Wote Amelo, Eyasu Makonnen

**Affiliations:** ^1^Department of Pharmacology and Clinical Pharmacy, School of Pharmacy, Addis Ababa University, Addis Ababa, Ethiopia; ^2^Center for Innovative Drug Development and Therapeutic Trials for Africa (CDT-Africa), Addis Ababa University, Addis Ababa, Ethiopia; ^3^Department of Pharmacology and Toxicology, School of Pharmacy, Jimma University, Jimma, Ethiopia

## Abstract

Since 2000, a good deal of progress has been made in malaria control. However, there is still an unacceptably high burden of the disease and numerous challenges limiting advancement towards its elimination and ultimate eradication. Among the challenges is the antimalarial drug resistance, which has been documented for almost all antimalarial drugs in current use. As a result, the malaria research community is working on the modification of existing treatments as well as the discovery and development of new drugs to counter the resistance challenges. To this effect, many products are in the pipeline and expected to be marketed soon. In addition to drug and vaccine development, mass drug administration (MDA) is under scientific scrutiny as an important strategy for effective utilization of the developed products. This review discusses the challenges related to malaria elimination, ongoing approaches to tackle the impact of drug-resistant malaria, and upcoming antimalarial drugs.

## 1. Challenges in the Control, Elimination, and Eradication of Malaria

Malaria control, elimination, and eradication are the long-term agendas of malaria endemic countries (MECs). Currently, almost all endemic countries are working to eliminate it with the ultimate goal of making the world free from this killer disease. Achievement of this goal depends on effective and integrated implementation of both preventive strategies and case management [1]. However, antimalarial drug resistance, insecticide resistance, and other mosquitos' survival tactics are the critical challenges towards the goal. For decades, antimalarial drug resistance remained the greatest challenge for malaria control and has been documented for almost all antimalarial drugs in current use [2]. Furthermore, two-thirds of malaria endemic African countries have reported resistance against, at least, two insecticides [3, 4]. Moreover, mosquitoes have also adopted survival tactics where they feed earlier and outdoors as well as rest outdoors after feeding indoors, bypassing the effect of indoor residual spraying (IRS) and insecticide-treated bed nets (ITN) [5]. Changes in the environmental and climatic conditions coupled with financial uncertainties are additional threats towards the eradication goal. These challenges related to chemotherapy and vector control have enforced the malaria research community to look for an effective malaria vaccine as high priority. However, despite many potential candidates on development, the hope for an effective and widely available vaccine seems to be challenging due to several factors ranging from the complex nature of the parasite to the cost of the developed vaccine [6].

In order to deal with multi-drug-resistant parasites, Artemisinin-based Combination Therapies (ACT) has been recommended. However, an insufficient supply of affordable and quality-assured ACTs has opened the room for parallel circulation of falsified and substandard products—antimalarial drugs appearing among the top lists [7, 8]. The malaria research community has already witnessed the impact of such substandard treatment from the emergence of artemisinin resistance (ART-R) in Southeast Asian countries where, once up on a time, majority of Artemisinin (ART) on the market were substandard or falsified [9]. Irrational drug use including artemisinin monotherapy and poor compliance are additional challenges towards the eradication goal [10]. The proposed solution to maximize the compliance and reduce the risk of resistance is the Single Encounter Radical Cure and Prophylaxis (SERCaP) [11]. In SERCaP, the drug is expected to have multistage activities including liver and transmission blocking, relapse prevention, and long-lasting effects to provide prophylactic treatment and kill all the malaria parasites in the body in one dose [12, 13]. Development of these products and making them available to those in need is another challenge.

## 2. Ongoing Approaches to Tackle Drug-Resistant Malaria

The emergence of artemisinin resistance, its spread to Southeast Asia (SEA) countries and some resistance reports from African countries are the indicators of the current threat to malaria control and elimination effort [14, 15]. This man-versus-parasite arms race related to drug development and the emergence and spread of resistance against such drugs dictates an urgent need for new antimalarial agents [16]. As a result, many approaches including new drug discovery and old drug repurposing are being pursued to the development of new antimalarial drugs. Parallel to this, the malaria research community is also working to identify novel drug targets for new drug development [17, 18].

Several reasons have been forwarded as challenges, especially, for antimalarial drug discovery [19]. Given the large number of population in MECs taking the medication and the underdeveloped health care system, the drugs need to be well tolerated and safe in humans. These drugs also need to be orally administered in a nonhospital setting with a maximum of a three-day treatment and with once or twice a day dosing. Furthermore, new drugs are also required to be used in combination and they should be of low cost. Together with such prerequisites, the lack of commercial market also affected the number of drugs that reach to the market for neglected infectious diseases [20]. Precompetitive approaches have been suggested and being applied to overcome these challenges and make more drugs available [21].

### 2.1. Working on Existing Antimalarials

#### 2.1.1. Optimization of the Therapy

As has well been recognized from the ACTs, optimization of treatment regimen through either dosage modification or combined use of drugs with different mechanisms of actions, favors antimalarial activity [22, 23]. However, pharmacokinetic and pharmacodynamic profile and history of monotherapy (preexisting resistance) with partner drug to be used with ART are the critical challenges during the selection of drugs for combination [24]. Various treatment optimization options are being tested to fight against the threat of ART-R and maximize its lifespan.

One of the potential options is increasing the total dose given to patients, as has historically been done with several antimalarial drugs like chloroquine (CQ) [25]. Another alternative strategy, which is proved effective, is to maintain the same daily dose but extending the course of routine 3-day treatment to 5 or 7 days [14]. This prolonged course of treatment has been worked well for artemether–lumefantrine (AL), but the effectiveness of other ACTs needs to be evaluated. However, these extended regimens may lead to drug safety concerns as a result of accumulation of the partner drug in the physiological compartment, where it elicits adverse effects, and may necessitate availability of un-combined ARTs risking monotherapy [26]. Increased duration of therapy would also affect the treatment compliance and may contribute for the emergence of the resistant strains. Furthermore, modelling suggested that splitting the current single dose into two (twice-daily dosing) would provide increased antimalarial effect and capable of restoring the effectiveness of failing ACTs [27]. As there is no clinical evidence to support this, more information is needed.

Based on the experience from the combination drug treatment of other infectious diseases, in addition to working on the dose and treatment duration of the usual ACT combinations, triple ACTs is also under way [28]. In this case, drugs that generate inverse susceptibility on the same target, where resistance to one inhibitor can cause parasite hypersensitivity to another, are being combined together. This effect is observed with two slowly eliminated drugs (lumefantrine (L) plus amodiaquine (AQ) or piperaquine (PPQ) plus mefloquine (MQ)) and has led to clinical trials with the triple ACTs: AL plus AQ or Dihydroartemisinin (DHA)-PPQ plus MQ) [29]. These studies aimed to test for the effectiveness of the combinations in the elimination of both resistant and sensitive parasites as well as potentially block the emergence of resistant parasites to the combinations.

#### 2.1.2. Development of Analogs of Existing Agents

Knowledge of the chemistry of existing drugs and their active moieties is very important to develop analogs for further screening [30]. This strategy is a cost-effective approach and has been a profitable endeavour. So many drugs have been developed based on this approach including CQ and quinine (QN) analogs such as piperaquine (PQ)/pyronaridine (PN) and AQ/MQ, respectively, each of which are now components of ACTs in use or in advanced trial stages [31, 32]. Other products of this approach include Primaquine (PQ) analogs (Tafenoquine (TQ)), aimed at a radical cure for P. vivax [33] and ART analogs (ozonide OZ439), a synthetic endoperoxide for use in combination with a partner drug [34]. Furthermore, the effects of pyrimethamine, sulphonamides, and *atovaquone* on pyrimidine metabolism have led to the development of new compounds targeting dihydroorotate dehydrogenase (DHODH), DSM265, and dihydrofolate reductase *(DHFR), P218, - overcoming resistance issues of pyrimethamine* [35–37].

#### 2.1.3. Using Resistance-Reversal Agents

Another approach of effective antimalarial drug development is preventing resistance against the existing agents, which may likely be achieved using resistance-reversal agents. Many drugs including calcium channel blockers, chlorpheniramine and PQ, have been shown to reverse the resistance of *P. falciparum* to CQ [38, 39]. Despite the availability of such drugs, there are many obstacles to translate this approach in to practice such as determination of drug levels required to achieve the therapeutic effect as well as lack of clinical research in this area [40].

Another approach is to reintroduce drugs which have been out of use for many years. The basic assumption of this approach is the prevalence of resistant parasites will decline with time when the drug to which resistance has developed is replaced by another one. In this situation, drug-resistant parasites will be competitively driven off by sensitive parasites for they have become less fit as well as they will not get continued advantage of drug selection pressure [41]. During reintroduction, it is important to be cautious about the appearance of other compensatory mutations that may increase the fitness of the resistant parasites and re-selection or importation of drug-resistant parasites after re-implementation of the drug. Several studies have reported emergence of CQ sensitive parasites after its withdrawal from countries like Malawi [41] and Tanzania [42]. The full recovery of CQ-susceptibility will help develop CQ-based combination drugs as a possible future alternative to Sulfadoxine-Pyrimethamine (SP) for intermittent preventive treatment for pregnant women (IPTp), where the public health benefits of IPTp are declining due to SP resistance.

### 2.2. New Drug Discovery and Development

#### 2.2.1. Approaches for Antimalarial Drug Discovery

Besides repurposing, there are additional drug discovery approaches called rational targeting of essential parasite processes (target-based) and whole-cell phenotype screening for compounds that produce a desired effect on the whole cell of the parasite (cell-based) [43]. A phenotypic screen focuses on identifying new chemical entities by phenotypic screening against whole cells, whereas a target-based approach requires validated and clearly defined targets. Some new antimalarial compounds that have been identified through cell-based approach include KAI407, KAF156, *Plasmodium* phosphatidylinositol-4-OH kinase inhibitors, KAE609, *P. falciparum* P-type ATPase 4 inhibitors, and DDD107498 [44–48]. The target-based approach has currently led to the discovery of *Plasmodium* DHODH inhibitor, DSM265, a compound with multistage antimalarial activity and the *inhibitor of DHFR, P218* [49].

Phenotypic-based screening is successful in identifying numerous new hit compounds but it provides little or no information on compound mode of action, thereby making it difficult to monitor emergence of drug-resistant parasites. However, it is still more preferred than the target-based approach for two reasons. On the one hand, it may result in discovery of compounds with many targets and lower chance of encountering resistant parasites than a single target-based approach. On the other hand, there is no need to work on the difficult task of target validation [13, 50]. The limited utilization of the target-based approach is more a result of lack of high-quality targets than the approach itself. However, recent advances in genetic understanding of *Plasmodium* have greatly increased the ability to genetically validate potential drug targets [17, 51], Identification of these targets will facilitate the discovery and development of drugs with novel mechanisms of action with less risk of cross resistance [52]. In general, we cannot discard the target-based approach but at the same time cannot rely only on a single target ignoring the complexity of cell [50].

#### 2.2.2. Novel Validated Targets for Antimalarial Drug Discovery

Currently, several crucial and unrelated biochemical pathways or enzymes present in malaria parasites have already been explored as drug targets [53, 54]. The reasons to look for such new targets include production of chemically diversified antimalarial drugs, which are less prone to cross-resistance, and generation of more number of effective and safe compounds through these targets [55, 56]. So, discovery of enough number of novel validated drug targets with simultaneous advancement of chemistry resources, necessary to produce appropriately selective and potent small molecules to test therapeutic hypotheses, is crucial to successfully develop new drugs effective against resistant parasites [17, 57].

There are two approaches for target validation, chemical and genetic [58]. In chemical validation, despite the difficulty to demonstrate selectivity of the inhibitor to a given target for the observed effect, it should usually show antimalarial activity *in vitro* and/or *in vivo.* The limitation of chemical validation can be solved by conducting parallel genetic validation, which focuses on determination of the effect of the corresponding gene deletion on the parasite's growth or survival. Then, the importance of that particular target will be defined through phenotypic analysis of the resultant mutant parasites in different phases of developmental cycle of plasmodium. Then suitable assays will be created to identify compounds (hit molecules) that have a desired activity at the drug target [59].

There are numerous criteria that must be met by a putative target to warrant investigation and to receive attention for compound testing. Some of the important characteristics include specific to the pathogen and part of a rate-limiting biochemical process; essential and have unique function; can be inhibited; and low potential for resistance development [55]. However, if the structural differences are well defined, targets that are shared between the parasite and human host can also offer opportunities for chemotherapy. For instance, the dihydrofolate reductase inhibitors, pyrimethamine and cycloguanil – the in vivo metabolite of Proguanil – are important components of antimalarial drugs [53]. Many drug targets can be related to the functions of distinct structures of the parasite. Of particular interest in this review are the recently discovered as well as revitalized targets, especially those which are clinically validated.

**Figure 1:**
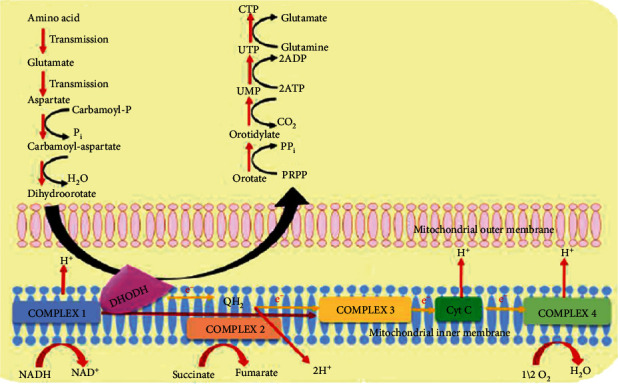
The working mechanism of *Pf*DHODH in *de novo* pyrimidine biosynthesis [61].

*(1) Dihydroorotate Dehydrogenase (DHODH)*. Malaria parasites derive their pyrimidine nucleotides only through a de novo pathway. This biosynthetic pathway requires properly functioning dihydroorotate dehydrogenase (DHODH), which is localized in the mitochondrial electron transport chain (mETC) [60]. DHODH catalyse the oxidation of dihydroorotate to orotate during the pyrimidine biosynthesis as shown in Figure 1. Pyrimidine is essential for the malaria parasite survival and its inhibition will be lethal. Despite its presence in both human and the *P. falciparum*, number of selective *Pf*DHODH inhibitors have been identified thereby exploiting the small difference between the binding pocket of the *P. falciparum* and human enzyme [61]. Among them, the lead compound, DSM265, has proceeded in to Phase IIa clinical trial [33].

*(2) Targets in Folate Metabolic Pathway*. *Plasmodia* can use either of two pathways, *de novo* or salvage, for the synthesis of tetrahydrofolate, a key coenzyme in amino acid and nucleotide metabolism. Successive steps in recycling folates for use in synthesis of thymidylate, purines, and methionine are catalysed by dihydrofolate reductase (DHFR) and thymidylate synthase (TS). Unlike in higher eukaryotes, in *Plasmodia*, DHFR and TS coexist as a single-chain bi-functional enzyme [62]. Two classes of antifolates, dihydropteroate synthase (DHPS) inhibitors (Sulfadoxine) and dihydrofolate reductase (DHFR) inhibitors (pyrimethamine) have been used in malaria treatment. Resistance to these antimalarials is conferred by dominant mutations in catalytic sites and/or amplification of the *Pfdhps* and *pfdhfr* genes and as a result of resistance to individual DHFR and DHPS inhibitors, the combination has also lost its effectiveness [63].

Among the mutations identified from clinical isolates of the parasite resistant to pyrimethamine, four point mutations (N51I, C59R, S108N, and I164L) in the DHFR domain portion of the DHFR-TS gene have been shown to be highly relevant to pyrimethamine resistance [64, 65]. In order to overcome such resistance and design inhibitors with novel binding modes, currently, researchers are working on the mutated versions of the enzyme. This effort has finally led to the discovery of the compound P218, which binds to the active site of *Pf*DHFR with a substantially different fashion from the human enzyme. Furthermore, in contrast to pyrimethamine, it binds both wild-type and mutant *Pf*DHFR thereby fitting well within the DHF substrate envelope [63]. The compound has recently proceeded into Phase I clinical development stage [33].

*(3) P. falciparum P-Type Cation ATPase (PfATP4)*. Spillman and Kirk (2015) have identified the role of the *Plasmodium falciparum* P-type ATPase 4(PfATP4) through phenotypic screening aiming at the generation of resistant parasite with the prolonged exposure to the compound of interest. PfATP4 is a parasite plasma membrane ATPase that regulates homeostasis thereby mediating active extrusion of Na + from the parasite to maintain low-[Na+]/high-[K+] in the cytosol. Inhibition of this target causes an increase in the parasite's cytosolic Na + level and the resulting osmotic water uptake leads to parasite death. Additional benefit of this target is its accessibility on parasite plasma membrane so that the drugs do not need to pass through the membrane.

Following the discovery of the first spiroindolones compound, KAE609 [45], targeting PfATP4, later phenotypic screens displayed the convergence of multiple chemical classes like aminopyrazoles, pyrazoleamides, and dihydroisoquinolones, upon PfATP4 [66]. This highlights the significance of this protein as a potential target for new generation antimalarial agents. From specific compounds identified for further development (KAE609, PA21A092, SJ733 etc.), KAE609 has progressed through Phase I and II clinical trials [66]. KAE609 displayed high activity against all asexual stages and also against gametocyte and oocyst development in mosquitoes, which is important to block malaria transmission [45].

**Figure 2:**
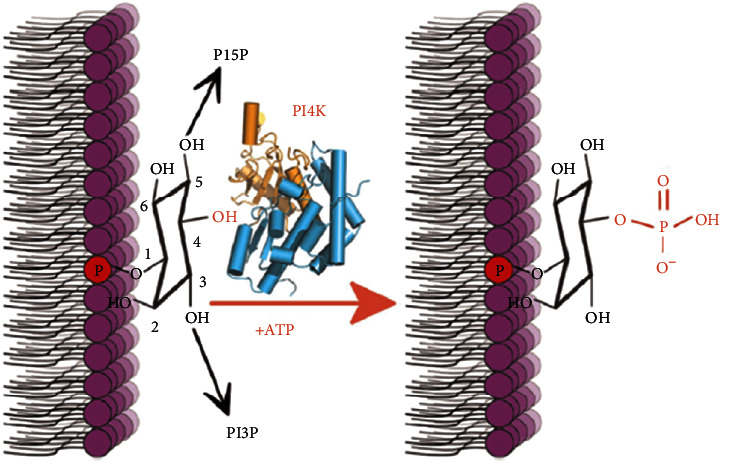
Schematic layout of the phosphorylation reactions at the inositol ring [68].

*(4) Phosphatidylinositol-4 Kinase (PfPI4K)*. Another new antimalarial drug target operating in all stages of the Plasmodium life cycle is phosphatidylinositol-4-kinase (PI4K). PfPI4K is a ubiquitous eukaryotic enzyme that phosphorylates lipids, at position 4 of the inositol ring, and subsequently synthesizes phosphatidylinositol 4-phosphate (PI4P) as depicted in Figure 2. Then the PI4P defines the membranes of Golgi and trans-Golgi network and regulates trafficking to and from the Golgi [45]. PI4P is a recruiter of lipid-binding effector proteins that are driven by Rab11A and required for generation of transport vesicles destined for the ingressing plasma membrane during merozoites biogenesis. It is important especially during the final step of parasite cell division, which requires delivery of new plasma membrane to the daughter cells [67]. PI4K, through production of PI4P, is, therefore, known to regulate intracellular signalling and trafficking. It is currently identified and clinically validated as an important drug target [44].

Imidazopyrazines were the first class of compounds identified as potent inhibitors of PI4K that inhibit the intracellular development of multiple *Plasmodium* species at each stage of infection. These compounds displayed potent preventive, therapeutic, and transmission-blocking activities in rodent malaria models as well as blood-stage field isolates. The activity of Imidazopyrazines is reported to likely stem from altered phosphatidylinositol 4-phosphate (PI4P) pools and disrupted Rab11A-mediated membrane trafficking because of the blockade of a late step in parasite development [44]. Subsequent search for additional compounds that inhibit PI4K enabled Paquet and colleagues (2017) to identify another chemical class, 2-aminopyridine, with potent activity. MMV390048, which was optimized from the class, displayed activity against multiple parasite life cycle stages. As a result of such activity, the compound was further developed, and currently, it is in clinical trials [33].

*(5) DOXP Reductoisomerase (PfDOXPR)*. A mevalonate independent isoprenoid synthesis pathway, the 1-deoxy-d-xylulose 5-phosphate (DOXP) pathway or methylerythritol phosphate (MEP), which is probably located in the apicoplast of plasmodium falciparum, is essential for the production of isoprene derivatives and shown to be a potential target for malaria chemotherapy [69]. In this pathway, there is condensation of glyceraldehyde 3-phosphate and pyruvate to DOXP and its subsequent conversion to 2-*C*-methyl-d-erythritol 4-phosphate, which is catalysed by DOXP synthase and DOXP reductoisomerase.

The inhibition of one of the key enzymes of this pathway, DOXP reductoisomerase, with fosmidomycin topped with its absence in mammals makes this pathway one of the potential new drug targets with low toxicity [69, 70]. Currently, in combination with piperaquine, fosmidomycin is being developed as a non-artemisinin-based combination therapy. Despite transient changes in electrocardiogram with prolongation of the QT interval, the combination displayed high efficacy with acceptable safety and tolerance [71].

*(6) Cytochrome bc_1_ (PfCTYbc_1_)*. In erythrocytic *Plasmodium falciparum,* mitochondrial electron transport supports pyrimidine biosynthesis. During the biosynthesis, ubiquinone functions as the electron acceptor for dihydroorotate dehydrogenase (DHODH). Regeneration of ubiquinone should, therefore, be in place for the proper functioning of this essential enzyme in *Plasmodium* species. This metabolic function in turn requires active mitochondrial electron transport chain (mETC). Cytochrome bc1 complex provides oxidized ubiquinone to DHODH through catalysing the transfer of electrons from ubiquinol to cytochrome c as shown in Figure 2. Despite the high cost of the drug targeting cytochrome bc1, it is clinically proven target for *atovaquone* [72].

Molecular analysis of samples collected from recrudescent malaria cases [73–75] and molecular dynamics simulations [76] have indicated the link between one point mutation in the cytochrome *b*, Y268S, and atovaquone resistance. Such mutation is reported to cause late treatment failure thereby reducing hydrophobic interactions between Cyt bc1 protein complex and atovaquone. Following this there has been increased effort to identify alternative cytochrome bc1 inhibitors with limited or no cross-resistance with atovaquine and likely to be much less expensive. One of the potential drugs that has been reported to be selective and specific inhibitor of *P. falciparum* mitochondrial bc1 complex is decoquinate [77]. As compared with atovaquone, decoquinate adopts different mode of binding within the ubiquinol-binding site of the complex and thus, reported to have little cross-resistance with atovvaquone [78]. *ELQ-300 is also* another drug being developed as a long-acting injectable chemo-protective agent. This parasiticidal drug is reported to inhibit cytochrome *bc*_1_ complex of *Plasmodium* parasites and bypass resistant mutations *to atovaquone* [79, 80].

### 2.3. Other Approaches

The future goal of transmission blockade, elimination and eradication of malaria requires integrated approach involving improved diagnostics, vector control, vaccination, as well as chemotherapy and chemoprophylaxis [17]. In addition to making these tools available to the target population, to achieve eradication goal, there should also be strategies to ensure rapid uptake by the population, surveillance and timely response as well as continued financial and political commitment [3]. Here we will discuss about the current progress on vaccine development and other new approaches for deployment.

#### 2.3.1. Vaccine

On top of treatment with effective antimalarials and mosquito control measures, to realize malaria eradication agenda, it is unwise to exclude the role of malaria vaccine. As a result, malaria vaccines have long been a research priority and currently, several vaccine trials, targeting one or more stages of the parasite life cycle, are underway. Based on the stages to be targeted, the vaccines can be categorized as preerythrocytic vaccines, blood-stage vaccines and transmission-blocking vaccines [81]. There is a growing appreciation that vaccines combining multiple targets and stages will be required for achieving and sustaining elimination [3].

The first vaccine to successfully complete a Phase III clinical trial and get a positive regulatory assessment is the RTS, S/AS01 vaccine [81, 82]. It is a preerythrocytic vaccine for the prevention of clinical *P. falciparum* malaria and conducted in African children. During the trial, it was administered with or without booster and prevented a substantial number of cases of clinical malaria over a three- to four-year period in young infants and children. Administration of a booster dose was reported to enhance the efficacy of the vaccine. It displayed 50% efficacy for clinical malaria in children aged 5–17 months old; while only 30% in target population, infants. Febrile seizures and other safety signals (meningitis, cerebral malaria and all-cause mortality in girls) were identified during the Phase 3 trial [83].

This vaccine has demonstrated the possibility of malaria vaccine development and augments the strategic goals of updated malaria vaccine technology roadmap to be met by 2030. The goal is to have vaccines, by 2030, that can provide at least 75% protective efficacy against clinical malaria, reduce transmission of the parasite, and can be deployed in mass campaigns [84, 85]. To this end, many next-generation *P. falciparum* malaria vaccine candidates, targeting all stages of the parasite lifecycle, are in early-stage development with the most advanced in Phase 2 trials [81, 86].

#### 2.3.2. New Approaches to Deployment

Recently, there has been renewed interest in an old and often controversial approach called mass drug administration (MDA). It is also called targeted malaria elimination, targeted malaria treatment, or targeted parasite elimination [3]. Notably, MDA has played a crucial role in the control and elimination of certain prevalent neglected tropical diseases and currently, recommended for *falciparum* malaria to bring rapid reduction of malaria related morbidity and mortality through reduced transmission. In order to apply MDA, the following criteria are required to be in place: being close to interrupt transmission, vector control, effective surveillance, and access to case management are at high coverage, and importation of infection is minimal; or for malaria epidemics [87]. Predicted time for effective MDA is to continue over 2 years rather than 1 year, and at the time of year when the lowest transmission occurs [88].

According to WHO, MDA consists of the administration of a full therapeutic course of antimalarial medicine to every member of a defined population or person living in a defined geographical area (except for those for whom the medicine is contraindicated) at approximately the same time and often at repeated intervals [87]. Based on this definition, the availability of exceptionally safe drugs for administration to such asymptomatic and uninfected individuals and also drugs that bring effective blockade of transmission are the visible uncertainties with this approach [17]. Current trial uses a full course of DHA-PPQ given in 3 day treatments three times at monthly intervals with a single low dose of PQ (0.25 mg/kg), to abort gametocytemia. DHA-PPQ is safe and well tolerated as well as reliably provides at least 1-month post-treatment prophylaxis [26]. For instance, no clinical *P. falciparum* cases were observed for at least a year after application of three rounds of MDA (a three-day course of DHA-PPQ plus PQ) and the regimen was reported to be safe with high coverage in Cambodia [89].

However, rising PPQ resistance, PQ safety, and undefined first-trimester safety of products considered for MDA, which are being deployed to areas where pregnancies are not usually reported in the first trimester, may threaten the effectiveness of this approach [3, 26]. Furthermore, there is also potential hazard with the exposure of ACTs to already resistant parasite population, which may lead to increased selection pressure with fatal consequences [3]. Hence, more work is needed in determining the most appropriate MDA regimen, the benefit of adding single dose PQ or TQ as well as potential incorporation of Ivermectin (IVM), anti-transmission agent. As it was evidenced from the reduction of the life-span of mosquitoes, which have ingested a blood meal from a person treated with IVM, integration of this drug in MDAs could make an impact on vector populations [90]. Additionally recommended approach to delay resistance and facilitate malaria control and elimination strategy is the deployment of different first-line therapies at the same time. This recommendation is based on the fact that the parasite that develops resistance against one drug will get eliminated when exposed to another drug in different host [26].

## 3. New Products in the Antimalarial Pipeline and Challenges Ahead

### 3.1. New Products in the Pipeline

Infectious nature of *Plasmodium* as well as emergence and spread of resistance against the parasite underlines the urgent need for continuous pipeline of new medicines with novel mechanisms of action [58]. On top of their effectiveness against emerging resistant strains of the parasite, although 3 days is the minimum acceptable regimen, these drugs, in order to improve adherence, need to be drugs with simpler courses of treatment allowing a single-dose cure [3]. Furthermore, to realize the malaria eradication agenda, the new drugs are also expected to block transmission, protect against infection and stop the relapse of *P. vivax.* Despite the long time it takes to make them available to the market, several compounds that can be partnered with other drugs in combination therapies are being rigorously tested and are progressing through the pipeline currently [14, 33, 91].

The evidence reporting Phase II trial result of KAE609, a new medicine from novel class of antimalarials called spiroindolones, has opened new milestone in antimalarial drug research [92]. Currently, many new products are under development, and majority of them are in partnership with Product Development Partnerships (PDPs). There are many products in the pipeline, which includes products for surveillance, prevention and treatment of malaria [3, 33, 91]. Among the products are the new medicines from Medicines for Malaria Venture (MMV) portfolio, which are on different clinical trial phases. Such compounds include those in phase I (MMV048, SJ733 and P218), II (OZ439, KAE609, KAF156 and DSM265) and III (TQ) [33, 93].

All new medicines in phase I and II are blood schizonticidals; all but DSM265 have transmission blocking activity and the majority have chemo- protective potential [33]. Those in phase II clinical trial have displayed activity in patients [49, 92–96]. They are relatively fast-acting compounds with long half-lives; as a result, a single dose of these drugs may potentially give coverage for over a week [3]. TQ lacks blood schizonticidal activity but it prevents relapse and bring radical cure in *P. vivax* malaria [33].

Beyond discovering new and effective drugs, there is another challenge of identification of best possible partner drug to combine with the new one. Extreme potency and tolerance required from such a SERCaP combination products makes the problem tougher [97]. Currently, there are three approaches that have been forwarded and being considered in the drug development pipeline. These approaches include use of existing partner drugs (PPQ), new members of the old families (ferroquine or PN) and two new drugs together [3]. Prior knowledge of degree of resistance and the risk of cross resistance among the combinations is very crucial. Some of these new drugs with established activity in clinical malaria cases are discussed here.

Despite the threat related to ART-R, there is still considerable interest for endoperoxide compounds. This is mainly dictated by the clinical success of ART and the longer duration of the action of these compounds, which could potentially counteract the K13-mediated ART-R [98]. As a result, two ozonides, namely, OZ 277 (arterolane) and OZ 439 (artefenomel), have been developed [33]. Arterolane is already licensed for clinical use in India as a fixed dose combination (FDC) with PPQ phosphate [99]. Artefenomel (OZ439) is a novel synthetic endoperoxide with suggested efficacy as a single encounter cure in combination with PPQ phosphate [94]. However, a phase II clinical trial reported that this combination failed to provide sufficient exposure for a sufficient duration to achieve the required efficacy as a single encounter treatment. There is also concern about cross resistance between artefenomel and ART, and probable coexistence of ART and PPQ resistance [100, 101]. Poor tolerability of artefenomel plus ferroquine combination has led to termination of the study. However, the rights of ferroquine has been transferred from Sanofi to MMV to further investigate in new Phase II combination studies [91]. Ferroquine, a new 4-aminoquinoline, has been observed to have no cross resistance with related drugs like CQ, AQ and also PPQ [102]. A new endoperoxide, tetraoxanes, with fast and long-lasting effect (including ART-R parasites), have also been reported to be a good alternative for ART [103].

KAF156 (Ganaplacide) belongs to imidazolopiperazines class of antimalarial molecules and brings about rapid clearance of both *P. falciparum* and *P. vivax* parasites in patients [95, 96]. KAF156, a new medicine whose mechanism of action is still being characterized, but may be related to a previously uncharacterized gene - *Plasmodium falciparum* cyclic amine resistance locus (*Pf*carl), is active against both asexual and sexual blood stages as well as the preerythrocytic stage of the parasite [96]. Currently, this molecule in combination with lumefantrine is under phase IIb trial as a once-daily administration for 3 days in different patient groups including children [91]. There is also other ongoing trials of this compound in combination with two new products, OZ439 and KAF156 [33].

DSM265, a triazolopyrimidine-based inhibitor of DHODH and the first inhibitor of this enzyme to reach phase II clinical trial, is also reported to be safe and efficacious, in a single dose, against both hepatic and blood schizonts of *Plasmodium falciparum* [33, 49]. A daily dose of a new synthetic antimalarial spiroindolones analogue, KAE609 (cipargamin), given for 3 days, was also observed to clear parasitemia in adults with uncomplicated *P. vivax* or *P. falciparum* malaria through inhibition of PfATP4. It has the shortest parasite clearance time (<1 h) than any other antimalarials discovered so far [92]. Cipargamin is being developed as the novel injectable compound for treatment of severe malaria [91]. Modified 4-aminoquinolones, AQ13, is another new agent currently undergoing phase II trials in Africa. This compound is reported to be more active than CQ against CQ-resistant falciparum malaria but with similar pharmacokinetics to CQ and also displayed non-inferiority to AL [104].

The only investigational medicine that currently completed phase III studies is TQ, which has been studied by GlaxoSmithKline in partnership with MMV. TQ is the same 8-aminoquinoline scaffold of PQ that brings *radical cure though elimination of P. vivax hypnozoite* and also shares the risk of haemolytic side effects of PQ in case of glucose-6-phosphate dehydrogenase (G6PD) deficiency. Unlike 14 day regimen with PQ, it will offer a single-dose cure for the liver-stage of *P. vivax* infections when administered with a standard 3-day CQ or potentially an ACT treatment regimen [33]. MMV048, the inhibitor of PI4K [44, 105], was reported to be the active compound against both *P. falciparum* and *P. vivax* across all stages of the parasite lifecycle but discontinued due to its teratogenic effect [33, 91].

### 3.2. Challenges Ahead

Variety of factors compromised the utilization of previously developed antimalarials and also the current attempts to maintain the efficacy of ARTs. One of such challenges is the strict need for fixed dose combination, where modification of ART dose will lead to increase in partner drug dose resulting in increased toxicity and adherence issues. Extending the course of treatment would also raise adherence issue as well as increased cost. Furthermore, drugs to be partnered with ART are also coming up with their own problems like toxicity, low efficacy and cross-resistance. Risk of hepatotoxicity with PN and low efficacy of PN–artesunate combination has cast doubt about the usefulness of the regimen as universal first-line treatment [106, 107]. Despite the difference in the degree of resistance, resistant strains have emerged against PPQ, LF, AQ, and MQ [108]. From the very beginning, the use of halofantrine (HF) was limited as a result of its cardiotoxicity [109] and *atovaquone,* on the other hand, failed to become a first-line treatment probably due to high cost and immediate emergence of resistance [110].

These flaws of earlier antimalarial drugs are, currently, raising the question about the promise of new candidates in the pipeline. Beside the history, there are also current lines of evidence to be sceptical about these products. For instance, there is a risk of earlier loss of KAE 609 and KAF 156 because of their low genetic barrier [41, 98] and untoward hepatic effect [111, 112]. Ozonides, arterolane, and artefenomel share peroxide pharmacophore with ARTs and this may risk cross-resistance within the artemisinin derivatives. There is recrudescence reports linked to resistant parasites against DSM265 and its partial activity against P. vivax parasites [113]. There are safety concerns evidenced with prolongation of QTc interval on administration of ozonides [94, 114] and elevation of creatine phosphokinase (CPK) and transaminases was also observed with Artefenomel [94]. Some of the compounds under development, for instance P218, has short half-life, necessitating daily administration to provide prophylaxis. To date, none of the formulations containing P218 have been able to reach the current target product profile. Finding appropriate partner drug for candidate drugs and the issue of single dose treatment, which might lead to the exclusion of valuable new treatments, are additional challenges ahead.

## 4. Conclusion

In the face of resistance against first-line antimalarial drugs and other related bottlenecks, sustained effort in developing new malaria tools is critical to meet the goals of malaria elimination and eradication. The current portfolio on development of the tools seems healthy and on good progress. However, time will tell how many of these products will effectively reach to the world's poor and contribute for malaria eradication. In general, unless complemented by such new products, the current interventions might be hardly enough to eradicate malaria.
